# Structural and Functional Disparities within the Human Gut Virome in Terms of Genome Topology and Representative Genome Selection

**DOI:** 10.3390/v16010134

**Published:** 2024-01-17

**Authors:** Werner P. Veldsman, Chao Yang, Zhenmiao Zhang, Yufen Huang, Debajyoti Chowdhury, Lu Zhang

**Affiliations:** 1 Department of Computer Science, Hong Kong Baptist University, Kowloon, Hong Kong SAR, China; wpveldsman@comp.hkbu.edu.hk (W.P.V.); cschaoyang@comp.hkbu.edu.hk (C.Y.); zmzhang@comp.hkbu.edu.hk (Z.Z.); 2 BGI Research, Shenzhen 518083, China; huangyufen@genomics.cn; 3 School of Chinese Medicine, Hong Kong Baptist University, Hong Kong SAR, China; debajyoti@hkbu.edu.hk; 4 Computational Medicine Laboratory, Hong Kong Baptist University, Hong Kong SAR, China; 5 Institute for Research and Continuing Education, Hong Kong Baptist University, Shenzhen 518057, China

**Keywords:** viral genome assembly, metagenomics, functional genomics, structural genomics

## Abstract

Circularity confers protection to viral genomes where linearity falls short, thereby fulfilling the *form follows function* aphorism. However, a shift away from morphology-based classification toward the molecular and ecological classification of viruses is currently underway within the field of virology. Recent years have seen drastic changes in the International Committee on Taxonomy of Viruses’ operational definitions of viruses, particularly for the tailed phages that inhabit the human gut. After the abolition of the order Caudovirales, these tailed phages are best defined as members of the class Caudoviricetes. To determine the epistemological value of genome topology in the context of the human gut virome, we designed a set of seven experiments to assay the impact of genome topology and representative viral selection on biological interpretation. Using Oxford Nanopore long reads for viral genome assembly coupled with Illumina short-read polishing, we showed that circular and linear virus genomes differ remarkably in terms of genome quality, GC skew, transfer RNA gene frequency, structural variant frequency, cross-reference functional annotation (COG, KEGG, Pfam, and TIGRfam), state-of-the-art marker-based classification, and phage–host interaction. Furthermore, the disparity profile changes during dereplication. In particular, our phage–host interaction results demonstrated that proportional abundances cannot be meaningfully compared without due regard for genome topology and dereplication threshold, which necessitates the need for standardized reporting. As a best practice guideline, we recommend that comparative studies of the human gut virome always report the ratio of circular to linear viral genomes along with the dereplication threshold so that structural and functional metrics can be placed into context when assessing biologically relevant metagenomic properties such as proportional abundance.

## 1. Introduction

Viruses evade classification by virtue of their minuscule size and vast diversity. Two years ago, the International Committee on Taxonomy of Viruses (ICTV) abolished the concept of a single type species, instead defining a species as a monophyletic group with multiple properties that distinguish it from other monophyletic groups in the same genus [1]. One year later, the ICTV abolished three major morphologically defined tailed phage families (Podoviridae, Siphoviridae, and Myoviridae) as well as the order Caudovirales within which they were contained [2]. The change was made in response to the outcome of multiple independent assessments that concluded that morphology-based virus families are polyphyletic with disparities in shared evolutionary histories. This was not the first time that a taxonomic order meant for tailed phage membership was disbanded (see the discussion of phage classification and the 50-year-old redundant Urovirales order in [3]).

Structure, however, remains a property of epistemological value as *form follows function*. The latter aphorism, which originated in the field of architecture but subsequently spread to other scientific disciplines including biology, alludes to the pivotal role that structure plays in our understanding of reality. Another point to bear in mind is that viral anatomy is decidedly different from that of other organisms due to the minimalistic nature of viruses. Like macromolecules, such as lipids and carbohydrates, viral features that are commonly considered morphological (e.g., the capsid and tail) are measured on the nanoscale. If these quasi-morphological features that delineate viruses from the outside world were to somehow be removed, all that would remain is the viral genome. The structure (or topology) of the viral genome would thus become a singular source of structural information. Despite phage genome sequences being much less conserved than phage structural proteins [4], sequence-based phage classification is often preferred to structure-based phage classification.

The distinction between circularity and linearity of phage genomes has not received the attention it deserves since, as late as 1998, an eminent review of the tailed phage literature stated that “The genome of tailed phages is typically a single molecule of linear dsDNA” [3]. The importance of genome topology is in part recognized by the Baltimore Classification System (BCS) [5] under the single- or double-stranded nucleic acid attribute. Nonetheless, the BCS has never considered whether the viral genome is circular or linear. There are, however, ample examples in the literature of biochemical studies on polynucleotide strands that can serve as support for the argument that viruses should be classified by circular and linear genome topology. For example, circular and linear DNA have been shown to differ in their mechanism of cytoskeletal transport [6], anisotropy [7], and structural transition (as discussed in [8]). These proven biochemical distinctions between circular and linear DNA strongly suggest biologically relevant distinctions between circular and linear viral genomes. Taking the latter suggestion from the field of biochemistry as a working hypothesis, we chose both extrinsic and intrinsic biologically relevant properties of viral DNA sequences as a basis for comparing circular and linear viral genomes. After considering typical measurements of interest in microbial analysis, we chose phage–host interaction, cross-reference functional annotation, and taxonomic classifiability as extrinsic properties, while we chose gene content, nucleotide frequency, dinucleotide skew, point and structural variation, and assembly quality as intrinsic properties. Moreover, our approach was multi-faceted in that we considered the preceding properties as dependent variables of molecular relatedness. In this manner, we could determine their values at intervals of average nucleotide identity (ANI) to elucidate trends in biological interpretability during increasingly stringent rounds of representative virus selection.

The process of representative selection is in essence a clustering exercise in which viruses are grouped together based on a predefined genomic similarity criterion. Throughout our paper, we refer to this clustering exercise as dereplication. At the strain level, no clustering is required. At the species level, 95% ANI is commonly considered appropriate [9]. However, the genus and family ANI thresholds vary widely and can fall anywhere between 50% and 95% [10,11,12,13]. With this non-standardized approach to delineating taxonomic boundaries at the genome-wide level for viruses, it is easy to appreciate the difficulties researchers encounter [14] when applying gene-level phylogenetic techniques that were honed on more evolutionarily stable genomes of living organisms. Moreover, these shortcomings in sequence analysis suggest that structural information should play a prominent role in virology. Considering that circularity versus linearity is not employed as a source of distinction by the major viral classification systems, and that there are no clear-cut ANI thresholds that define a virus taxon, a natural question arises: what impact do viral genome topology and dereplication thresholds have on structural and functional annotation? The results of our study, which was aimed at addressing this question, show that genomes classified by topology and dereplication stringency differ remarkably in terms of genome quality, GC skew, transfer RNA (tRNA) gene frequency, structural variants (SVs), cross-reference functional annotation (Clusters of Orthologous Groups [COG], Kyoto Encyclopedia of Genes and Genomes [KEGG], Protein Families Database [Pfam], and The Institute of Genomic Research Functional Analysis and Classification of Proteins [TIGRfam]), state-of-the-art marker-based classification, and phage–host interaction. Based on these findings, the best practice we recommend is that comparative viromics studies of the human gut genome always report the ratio of circular to linear viral genomes (ΔCL) along with the dereplication threshold so that molecular (e.g., gene frequency) and ecological (e.g., phage–host interaction) metrics can be accurately compared.

## 2. Methods

### 2.1. Source of Human Gut Metagenomic Sequencing Reads

We relied on metagenomic sequencing datasets that were created in a previous study of genetic variation within the human gut microbiome [15]. These datasets were deposited at the National Institute of Health’s sequence read archive under BioProject PRJNA820119. We downloaded long-read datasets of 200 Chinese individuals that were generated using an Oxford Nanopore Technology (ONT) PromethION platform via the EMBL-EBI FTP server (ftp.sra.ebi.ac.uk; accessed on 22 May 2023). We also downloaded 200 matching short-read datasets (150-bp paired-end reads) generated using an Illumina NovaSeq platform via the same EMBL-EBI server. Short reads were obtained with the purpose of polishing assembled viral contigs. Summary long- and short-read statistics and plots were generated using NanoPlot v1.41.0 [16] and fastp v0.23.4 [17] to ascertain and compare the quality of the ONT long-read and Illumina short-read sequences.

### 2.2. Viral Genome Assembly, Genome Dereplication, and Genome Quality Ascertainment

Viral genomes were assembled using viralFlye v0.2 [18], which requires contigs specifically generated by metaFlye as input [19]. We first passed raw ONT reads to the metaFlye v2.9.2-b1786 assembler using the nano-raw flag. To determine the assembly approach that would lead to the largest number of assembled viral genomes, we benchmarked the viralFlye assembler by permuting the use of (i) the metaFlye meta flag, which is used to correct for uneven read coverage; (ii) internal short-read polishing using viralFlye; and (iii) external long-read polishing using Medaka v1.6.1 (designed by ONT). To remove technical replicates that arise from repeated viral assembly for each of the 200 samples and to select representatives of the lower taxa, we carried out dereplication using the cluster functionality in MMseqs2 v14.7e284 [20] with relaxed overlap calling (--cov-mode 1 -c 0.01). Another clustering program called dRep [21] is commonly used in microbial genome clustering; however, the authors of dRep state that virus genome clustering with their software requires the use of an independent genome completeness estimator. To promote genome completeness in our approach, we explicitly set the MMseqs2 cluster mode flag to 2 in order to reduce the selection of shorter sequences as representative sequences. MMseqs2 automatically outputs representative sequences after clustering, and we used all output sequences as representative sequences in downstream analysis. Minimum sequence identity (--min-seq-id) was set to 0.95 to dereplicate to species level, 0.70 to dereplicate to genus level, and 0.50 to dereplicate to family level. A threshold of 95% is commonly chosen as a species-level cut-off (see [22] as an example) and is the Minimum Information about an Uncultivated Virus Genome (MIUVIG) standard for viral operational taxonomic units [9]. Values between 50% and 95% are more arbitrarily selected in the literature as taxonomic boundaries. We chose a relatively stringent interpretation of 70% for genus and 50% for family as the assembled viral genomes were recalcitrant to clustering at higher sequence identities. Summary viral genome statistics (including GC skew) were calculated using the fx2tab functionality in SeqKit v2.3.0 [23] and visualized using R 4.2.2 [24] with the library ggpubr v0.6.0 [25]. We assessed the quality of assembled viral constructs using checkV v1.0.1 [26]. The file containing the mean GC skew and mean GC content that were calculated for each short read in all 200 samples was downsampled to 10% of its original size using simple random sampling to carry out comparative statistical procedures with the computational resources at our disposal. We noticed that the shape of the GC skew distribution qualitatively differed between linear and circular sequences, and we therefore used empirical cumulative distribution function analysis, bundled in the R package twosamples [27], to quantify the differences.

### 2.3. Viral Genome Annotation, Taxonomic Classification, and Host Prediction

The viral genomes that were automatically classified as either circular or linear by the viralFlye assembler were functionally annotated using geNomad v1.5.2 [28] with its end-to-end pipeline—which includes a neural network implementation and custom viral profile database for the identification of proviruses and plasmids—for marker-based taxonomic classification and the functional annotation of viral genomes with cross-reference identifiers (COG, KEGG, Pfam, and TIGRfam). The most likely host for each assembled viral genome was predicted using iPHoP v1.3.2 [29]. tRNAs were detected using tRNAscan-SE v2.0.12 [30] with its general tRNA model selected as the tRNA detection model. SVs (insertions [INSs] and deletions [DELs]) were detected using Sniffles v2.0.7 [31,32] with preprocessing using minimap2 v2.26-r1175 [33] and SAMtools v1.17 [34]. We chose Sniffles because it detected a more diverse range of both real and simulated SVs (DELs, duplications, inversions, and INSs) than other long-read-specific SV callers during a 2019 benchmark study [35]. As read quality is especially important during variant calling, the long reads were filtered (q = 12, u = 5), trimmed (f = 10, b = 10,000), and deduplicated using fastp v0.23.4 [17] prior to mapping the reads to the viral genomes.

### 2.4. Protein Structure Prediction and Ortholog Detection

Determining protein orthology in terms of tertiary structure allows for the detection of remote homologs and analogs that are characterized by reduced sequence similarity. To supplement geNomad sequence-based viral gene and protein prediction, we carried out structural orthology analysis using FoldSeek v 7.04e0ec8 [36], which is a newly developed tool capable of carrying out previously infeasible all-against-all comparisons of vast sets of tertiary protein structures. We first predicted the tertiary structures of the predicted viral protein sequences using application programming interface (API) calls to the evolutionary scale modeling (ESM) Metagenomic Structure Atlas [37]. The resultant protein structure files in Protein Data Bank (PDB) format were then compared with tertiary structures in the AlphaFold Protein Structure Database [38,39]. API calls to the ESM Metagenomic Structure Atlas did not robustly respond to requests. Structural orthologs were therefore not used in our comparison of topological and dereplication disparities, but solely to supplement sequence-based ortholog detection.

## 3. Results and Discussion

### 3.1. Viral Genome Assembly Quality and Provirus Detection

CheckV reported that the viral genomes that were assembled and polished using viralFlye were of good quality (Table 1). We followed a stringent approach in our quality assessment. We placed all viral genomes that were not deemed as high quality by both MIUVIG and CheckV standards into a low-quality category. Medium-quality viral genome assemblies were, therefore, also placed in the low-quality category. The majority of viral genomes (96%) had no detectable host integration signals with 84% of the latter being of high quality. The latter percentage of high-quality genomes remained consistent throughout dereplication. However, prior to dereplication, high-quality circular genomes without detectable integration signals were 3.3× more abundant than high-quality linear genomes. The ratio of high-quality circular to linear genomes, which we acronymize as ΔCL, decreased during dereplication to 2.1× at the species level and decreased further to 1.6× at both the genus and family levels. The opposite trend was seen for high-quality linear genomes that had detectable host integration signals. For the latter presumed proviruses, high-quality linear genomes outnumbered circular genomes by nearly fourfold, with the ratio increasing during dereplication.

### 3.2. GC Skew Is a Biologically Relevant Property in Topological Genome Conformation

The 200 ONT long-read datasets from the study by Chen et al. contained a combined total of 147.3 million reads with approximately a third of the reads having a quality score in excess of Q12 (Supplementary File S5). The long reads also had a mean GC content of 45.3% and a mean GC skew of +0.13, indicating a slightly higher mean abundance of guanine than cytosine.

Fastp analysis of the Illumina short reads confirmed that the read adapters had previously been trimmed and that 92.5% of the 11.2 billion reads (forward and reverse) across the 200 samples had a quality score in excess of Q30 (PHRED), likewise indicating previous quality filtering (Supplementary File S6). The short reads had a mean GC content of 46.8% and a mean GC skew of +0.22, which, as in the case of the long reads, indicates a slightly higher mean abundance of guanine than cytosine.

We determined that the best assembly approach to follow was viralFlye internal short-read polishing with the meta flag activated during the prerequisite metaFlye step (Table S1). The latter approach remains advantageous when considering the total number of genomes (circular and linear) retained after all of the three dereplication rounds, that is, at a minimum sequence identity of 0.95 at the species level, 0.70 at the genus level, and 0.50 at the family level. However, after first and second rounds of dereplication, more circular viral genomes were obtained when not using viralFlye internal short-read polishing. Nonetheless, in both these subcases, our selected approach performed second best out of the five tested approaches, leaving our selected approach as the best approach in six of the eight subcases (see “Most genomes in dereplication category” column in Table S1).

Statistical tests for normality of the mean GC content and mean GC skew of both the long reads and short reads revealed that not one of the four respective vectors was normally distributed (Anderson–Darling test, *p* << 0.05), and that the GC content deviated at least 10 times more from normality than did GC skew (as indicated by the Anderson–Darling test statistic). We accordingly tested for homogeneity of variance using a non-parametric test that is also robust against differences in the sample size. The variance of both the GC content and GC skew differed between long and short reads (Fligner–Killeen test, *p* << 0.05), but, in contrast to the greater departure from normality that was seen in the GC content during the tests for normality, the greater departure from equal variance was between the GC skew of long reads and the GC skew of short reads (as indicated by the Fligner–Killeen test statistic). These tests served to empirically confirm that short reads have a higher frequency of guanine than long reads, perhaps as a result of differing accuracies between long and short reads. Short reads are widely known to be more accurate than long reads, which leads to differences in genome assembly quality. With these statistics on metagenomic reads, we next analyzed GC skew profiles in the assembled viral genomes.

In total, 1485 viral genomes were assembled with the viralFlye assembler using our selected approach (Table 2). Roughly two thirds of the viral constructs were circular, and the rest were linear. As mentioned earlier, the ratio of circular to linear constructs decreased during dereplication, but circular constructs remained the most abundant. In contrast, the average length of viral genomes increased during dereplication (except after the first dereplication round), which suggests that manual adjustment of the MMseqs2 cluster-mode parameter promoted the selection of longer representative viral genomes, as expected.

**Figure 1:**
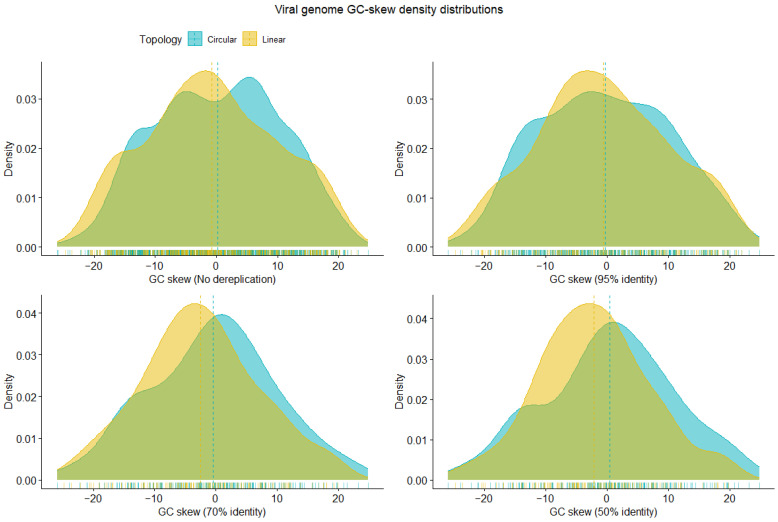
Importance of GC skew in viral genome topology. (Top left to bottom right) Circular and linear genome GC skew prior to dereplication and after 95%, 70%, and 50% sequence similarity clustering. The shapes of the circular and linear GC skew density distributions are noticeably different at each dereplication level. Empirical cumulative distribution function analysis (α = 0.05) to determine whether circular and linear viral genome GC skews come from the same distribution revealed that the two distributions are significantly different at 50% identity (*p* = 0.017), somewhat nonsignificantly different at 70% identity (*p* = 0.111), nonsignificantly different at 95% identity (*p* = 0.625), and only marginally nonsignificantly different prior to dereplication (*p* = 0.052).

The shape of the GC skew density distribution of circular viral genomes differed noticeably from that of linear genomes (Figure 1). Empirical cumulative distribution function analysis revealed that the probability that the circular and linear viral genome GC skew values were from different distributions was significant at 50% identity (*p* = 0.017), somewhat nonsignificant at 70% identity (*p* = 0.111), nonsignificant at 95% identity (*p* = 0.625), and only marginally nonsignificant prior to dereplication (*p* = 0.052). The mean GC skew of the 1485 genomes in the strain set was −0.09, while those of the dereplicated genomes were −0.48 in the species set, −1.41 in the genus set, and −0.71 in the family set. Circular viral genomes consistently exhibited a higher mean guanine abundance than linear genomes: strain set (circular: +0.23; linear: −0.73), species set (circular: −0.36; linear: −0.64), genus set (circular: −0.46; linear: −2.47), and family set (circular: +0.46, linear −2.12).

GC skew is known to be a non-trivial property that occasionally reflects the presence of certain genomic features. For example, a change of polarity (the sign) of GC skew indicates features such as the origin of replication [40] and the site of mobile genetic element insertion [41]. The consistently higher mean GC skew that we observed in circular viruses relative to linear viruses may play a role in structural configuration given that GC skew in this case is a property that discriminates between two topological classes. Considering that these viral genomes were all assembled with long reads using short-read polishing, it can be inferred that the difference in quality between long and short reads does not have bearing on the assembly of topologically different viral genomes exhibiting different GC skews. In other words, the difference in GC skew between circular and linear viral genomes cannot be an artifact caused by nucleotide base frequency and quality disparities between long and short reads. GC skew is therefore a biologically relevant property in topological genome conformation.

### 3.3. Circular Viral Genomes Contain More Trnas Than Linear Viral Genomes

**Figure 2:**
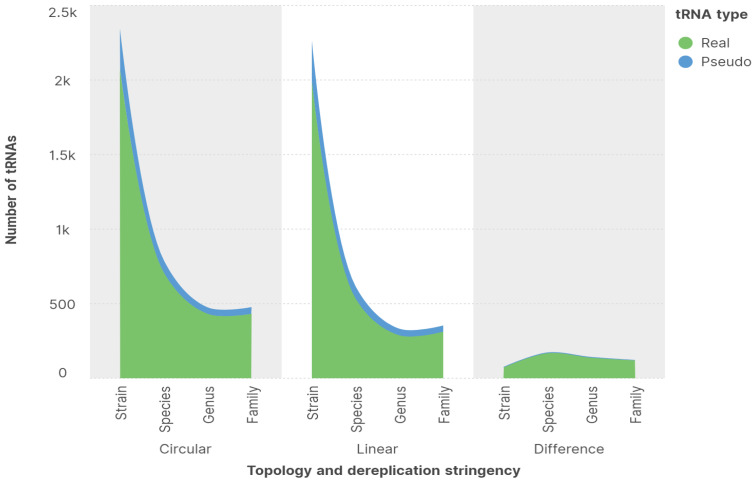
Transfer RNA frequency. Circular viral genomes contain a larger number of real (high-confidence) tRNAs than do linear viral genomes; however, the difference between circular and linear viral genomes in terms of pseudo-tRNAs is marginal. The majority of detected tRNAs are lost during strain to species dereplication, while the bias toward circular viral genomes increases at the same time.

Phage genomes are known to contain tRNA genes in greater abundance than any other genes involved in translation [42]. Furthermore, virulent phages contain more tRNAs than temperate phages, which implies that tRNA function goes beyond protein synthesis to impact the viral life cycle. We accordingly analyzed the occurrence of tRNAs in our assembled viral genomes to determine whether there are differences in the number of detected tRNAs between circular and linear viral genomes, and whether tRNA detection frequency is affected by dereplication. As more circular genomes were assembled than linear genomes in our study, we normalized the number of detected tRNAs. Despite the normalization step, we found that circular genomes had more detectable tRNAs than linear genomes on average (Figure 2). This bias toward circular genomes was constituted mostly by the number of tRNAs that were called with high confidence by tRNAscan-SE. The difference between circular and linear genomes in terms of the number of detected pseudo-tRNAs was trivial. A second observation was that the number of detected tRNAs decreased by approximately two thirds during strain to species dereplication, while the bias toward tRNAs in circular genomes more than doubled. The doubling of the aforementioned bias toward circular genomes decreased somewhat during second and third rounds of dereplication but remained nearly double the number observed prior to dereplication. This drastic initial increase in detected tRNA during strain to species dereplication was a pattern that we also observed during the detection of SVs (see Section 3.4) and taxonomic classification (see Section 3.6). From the similarity of these patterns, we deduced that tRNAs may interact with circular genomes in a strain-dependent manner as the patterns, as mentioned in Section 3.4 and Section 3.6, are Crassvirales-strain dependent. This deduction is supported by our observation that the ratio of tRNA-containing circular Crassvirales genomes to tRNA-containing circular genomes that were lost by clustering during strain to species dereplication was more than double the ratio of tRNA-containing linear Crassvirales genomes to tRNA-containing linear genomes that were lost during strain to species dereplication. To end our assay of tRNAs, we compared the compositional abundance of the anti-codons on the detected tRNAs (Table 3). We found that Met-tRNA was always the most abundant tRNA regardless of topology and dereplication set, while Val-tRNA was always the least common tRNA in linear viral genomes and His-tRNA (except in the strain set) was always the least common tRNA in circular viral genomes.

### 3.4. Dereplication Increases the Detection Rate of SVs

**Figure 3:**
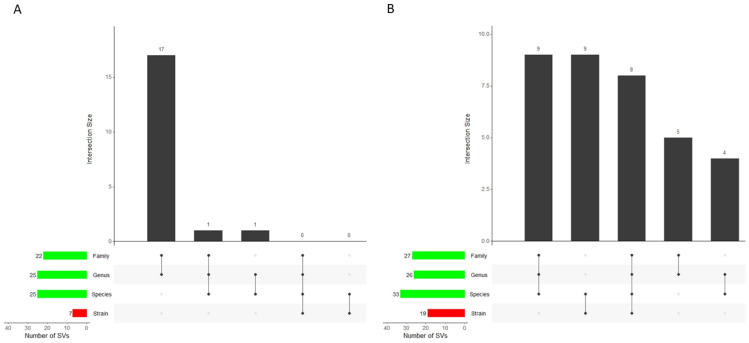
Structural variant frequencies in (**A**) circular and (**B**) linear viral genomes. Structural variants (SVs) are less frequent in the non-dereplicated strain sets than in the dereplicated sets despite the dereplicated sets containing fewer viral genomes. Only eight SVs appear consistently in all sets, with all eight detected in linear viral genomes. Note: The plots in panel A and B are analogous to Venn diagrams with the intersection size on the *y*-axis being the frequency of SVs (insertions and deletions).

Genes within virus genomes are tightly packed due to an intense natural constraint on viral genome size. Nevertheless, viral genomes are imperfect and undergo rapid mutation, which introduces not only the commonly known point mutations but also SVs. Research has shown that SVs affect viral plaque size and viral dissemination in a strain-dependent manner [43]. As species identification is a prerequisite for strain identification and by implication must be accompanied by some form of representative genome selection (a process known as dereplication), we sought to determine SV profiles (INSs and DELs) at the same dereplication levels that we compared elsewhere in the current study, that is, at the strain level, species level, genus level, and family level. In our analysis of tRNA profiles, we saw a pattern in which first-round dereplication (dereplication from strain to species level) was accompanied by a sharp increase in tRNA detection in favor of circular genomes, followed by a slight tapering off during subsequent dereplication. Here, in our analysis of SVs, a similar pattern emerged (Figure 3). We detected seven and nineteen SVs in the circular and linear strain sets, respectively. Upon species-level dereplication, the number of SVs more than doubled to 25 and 33, respectively. In our opinion, this result implies that SVs are part of the reference genomes at the strain level. Once representative species are selected by the process of dereplication, SVs are no longer identical to sequences in the species set and are flagged as variants. This is particularly noticeable in the circular sets where none of the seven SVs observed in the circular strain set was retained during the first dereplication round, while eight SVs from the linear strain set were retained not only during the first dereplication round but also in all of the dereplication sets (Table S2).

### 3.5. Viral Genome Topology and Representative Genome Selection Affect Functional Annotation

Four databases were cross-referenced for functional annotations: KEGG [44], Pfam [45], TIGRfam [46], and COG [47]. Analysis of the annotations per topology and per dereplication level indicated that viral genome topology and dereplication strategy have a major impact on functional annotation (Table S3). We used the top ten most frequent annotations as a metric to compare the relative frequency of annotations across different groups. Only three functional cross-references (“xrefs”) appeared consistently in the top ten annotations across topologies and across dereplication levels: TIGR01547 (phage terminase), TIGR00673 (cyanase involved in cellular detoxification), and COG1783 (phage terminase). A fourth xref, PF05133 (phage portal protein), appeared in seven of the eight strata. TIGR01725 (phage morphogenesis) and COG5005 (Mu-like prophage protein) entered the top ten xrefs during the first circular viral genome dereplication round, while two bacterial DNA primases (TIGR01391 and COG0358) dropped to 53rd and 78th positions, respectively. The second dereplication round promoted the relative frequency of annotation of PF13392 (HNH endonuclease) and PF03864 (phage major capsid protein E) while improving the position of the previously mentioned DNA primases (TIGR01391 and COG0358) to 43rd and 27th, respectively. The final round of circular genome dereplication only had the effect of internally shuffling the top 10 xrefs, likely because the difference in the number of genomes between the last round dereplication sets was not as great as the difference in the number of genomes between the first two dereplication round sets. A repeat of the analysis of linear genomes revealed that strain-level linear genomes shared only half of their top ten xrefs with circular genomes. The first round of dereplication of linear genomes promoted the importance of TIGR01633 (putative phage tail component) and COG4926 (phage-related protein), while further dereplication of linear genomes had a less pronounced impact on the top 10 xrefs. An example of the implication of these observed differences in the relative frequency of xref annotation is that, if a genome assembler is prone to assembling more circular genomes than linear genomes or vice versa, such technical properties of the assembler will propagate to functional analysis of the genomes, where it will have a non-trivial impact on biological interpretation regardless of whether the assemblies are correct. Similarly, the process by which a representative sequence is selected will also have a non-trivial impact on downstream biological interpretation, which in turn has implications for viral strain analysis.

### 3.6. The Vast Majority of Human Gut Viruses Are Tailed Phages That Defy Marker-Based Classification

More than 99% of the viralFlye assemblies were confirmed as viral by geNomad taxonomic classification. The confirmed virus percentage decreased to >98% during the first dereplication round and remained at that level for the remainder of the dereplication rounds. This decrease was not unexpected as non-viral representatives would necessarily be retained during dereplication. More than 98% of the viruses in the strain set belonged to the realm Duplodnaviria, which includes double-stranded DNA viruses that have a characteristic major capsid protein exhibiting an HK97 protein-fold. Here too, dereplication decreased the percentage of Duplodnaviria due to representatives of Monodnaviria and Riboviria viruses being detected. However, the percentage of Duplodnaviria remained above 96% in all sets, and, importantly, all Duplodnaviria viruses in all dereplication sets belonged to the class Caudoviricetes. The vast majority of Caudoviricetes were unclassifiable beyond the taxonomic rank of class (strain set = 88%; species set = 94%; genus set = 94%; family set = 93%). One reason why so many sequences were unclassifiable is that we used geNomad’s stringent taxonomic classification approach in which at least 50% of a custom weighted score must support a specific taxon for a taxonomic name to be assigned to a genome. Second, the 6% initial increase in unclassifiable genomes during strain to species dereplication can be explained by classifiable CrAss-like phages going from having 165 strain representatives to having only 29 species representatives, thereby increasing the relative number of unclassifiable sequences. Although there are ample examples in the literature of attempts at phage family classification [48], there is still no standardized approach to evidence-based classification of metagenomic viruses [14]. As the scope of our project was limited to determining the impact of genome topology and representative genome selection on taxonomic classification, we did not investigate the impact beyond the phylum and class taxonomic ranks, both of which are already clearly impacted by genome topology and dereplication. However, we suggest that a possible improvement in the limitations of geNomad’s MMseqs2-based protein-profile searches may lie in the use of tertiary protein structure comparison. As shown in the next section, tertiary structure prediction leads to the detection of plausible orthologs that challenge results derived from sequence-based ortholog detection.

### 3.7. The Feasibility of Phage–Host Comparative Studies Depends on the Availability of Strain Data

**Figure 4:**
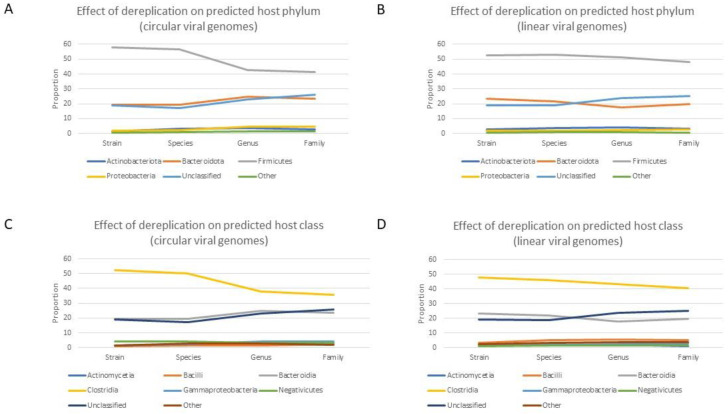
Predicted bacterial hosts. (**A**,**C**) Dereplication reduces the proportional difference between Firmicutes and Bacteroidota for circular viral genomes, while (**B**,**D**) the same proportional difference remains relatively stable during the dereplication of linear viral genomes. These graphs also clearly illustrate that the dereplication threshold determines the proportional abundances of predicted bacterial host taxa in the human gut, which would have a non-negligible impact on comparative viromics studies. Note: some lines are obscured at the bottom of the subplots due to overlapping proportional abundance.

Phages infect specific bacteria. The infection specificity is primarily determined by the specificity of adsorption, which correlates with specific receptors on the extracellular host surface (as discussed in [49]). However, once a phage attaches to a host, it must overcome a formidable molecular barrier to inject its DNA into the host cell. DNA topology confers physicochemical properties that may play a role in this regard (as exemplified by the sought-after properties of circular single-stranded DNA in theranostics [50]). We therefore investigated whether there is a difference between the predicted bacterial hosts of linear and circular viral genomes. In line with our findings on structural and functional features, our results here revealed that genome topology also discriminates between linear and circular viral genomes in terms of their predicted bacterial hosts. In our strain set, circular viral genomes had a larger proportional difference between Firmicutes and Bacteroidota (referred to hereafter as ΔFB) than linear genomes (Figure 4). Dereplication analysis showed that the ΔFB for circular viral genomes decreased during dereplication, with Firmicutes becoming less abundant and Bacteroidota becoming more abundant, while the ΔFB for linear genomes remained relatively stable albeit with lower abundances of both Firmicutes and Bacteroidota. The implication of the difference in ΔFB between circular and linear genomes is that robust comparisons cannot be made between metagenomic studies of the whole gut virome if there is no explicit indication of the nucleotide similarity threshold that was used during representative genome selection and of the ΔCL. The lack of robustness is compounded by circular and linear viral genomes undergoing different changes in their ΔFBs during dereplication. We furthermore noticed an exception to the lowering of the Firmicutes proportion during circular genome replication wherein the bacterial class Negativicutes (phylum: Firmicutes) was predicted more often as the host of phages with circular genomes than as the host of phages with linear genomes. The genus most frequently predicted as a host is *Bacteroides*, while the bacterial species most frequently associated with multiple high-confidence AlphaFold structural ortholog hits in the human gut virome is *Enterococcus faecium* (see data availability for protein models), a bacterium whose clinical and non-clinical strains have distinct structural and functional features [51], suggesting a cryptic bacterial species that diverged in the absence of in situ ecological relationships.

## 4. Conclusions

As viruses are nanobionts, drawing a line of distinction between which aspects of their study fall under morphology and which fall under molecular biological is challenging. Although the concept of a type species is no longer recognized by the leading authority in virology, the concept of a monophyletic group, which has replaced the type species, still requires shared molecular and ecological characteristics. In this study, we demonstrated that genome topology and representative genome selection have a non-trivial impact on biological interpretation. We report on the results of seven separate experiments to assay the difference between circular and linear genomes. Each experiment revealed a remarkable difference between circular and linear viral genomes in terms of not only molecular features but also the process by which representative viruses are selected. To allow for the accurate comparison of human gut viromes between studies, we recommend that researchers report the ΔCL along with dereplication thresholds. The ΔCL is limited in that it does not model exceptions to observed differences between circular and linear viral genomes, such as the fact that circular phages exhibit an overall decrease in the proportion of predicted Firmicutes hosts during dereplication, with the class Negativicutes being a notable exception. Nevertheless, the ΔCL provides a basis for due consideration of the structural and functional differences between circular and linear viral genomes and serves as a draft for future modeling of the proportional abundance of circular and linear viruses in the human gut.

## Figures and Tables

**Table 1:** Quality of the assembled viral genomes.

Dereplication Set	Topology		Total	ΔCL *	Quality	Integrated
	Circular	Linear				
Strain	938	281	1219	3.3	high	No
	47	167	214	0.3	low	No
	7	27	34	0.3	high	Yes
	3	15	18	0.2	low	Yes
Species	352	167	519	2.1	high	No
	22	79	101	0.3	low	No
	3	24	27	0.1	high	Yes
	1	9	10	0.1	low	Yes
Genus	165	105	270	1.6	high	No
	12	34	46	0.4	low	No
	2	16	18	0.1	high	Yes
	0	5	5	0.0	low	Yes
Family	158	99	257	1.6	high	No
	14	29	43	0.5	low	No
	2	12	14	0.2	high	Yes
	0	5	5	0.0	low	Yes

* The ratio of circular to linear genomes.

**Table 2:** Summary statistics of the assembled viral genomes.

Dereplication Set	Number of Genomes			Minimum Length (bp)		Maximum Length (bp)		Average Length (bp)	
	Linear	Circular	Total	Linear	Circular	Linear	Circular	Linear	Circular
Strain	490	995	1485	5446	5301	232,946	213,711	58,213	67,205
Species	279	378	657	5773	5301	232,946	213,711	56,041	60,639
Genus	160	179	339	7455	5525	232,946	213,711	62,142	71,731
Family	145	174	319	7455	5525	232,946	213,711	63,799	72,772

**Table 3:** Ranking of the predicted tRNAs by their relative abundance (including pseudo-tRNAs).

Rank	Circular									Linear							
	Strain		Species		Genus		Family			Strain		Species		Genus		Family	
1	Met	13.35	Met	13.43	Met	13.47	Met	13.84		Met	10.32	Met	11.59	Met	11.92	Met	11.86
2	Undet	11.05	Undet	10.52	Leu	9.47	Leu	9.43		Undet	9.70	Undet	10.23	Ser	8.28	Leu	7.46
3	Gln	8.45	Gln	8.62	Gln	8.00	Gln	8.39		Arg	8.47	Arg	7.27	Lys	7.28	Undet	7.46
4	Leu	8.36	Leu	8.37	Undet	8.00	Undet	7.55		Leu	7.58	Lys	6.82	Undet	6.95	Ser	7.46
5	Arg	7.21	Ser	7.22	Arg	6.74	Arg	6.92		Ser	7.14	Leu	6.59	Leu	6.62	Lys	7.12
6	Ser	6.61	Arg	5.45	Ser	6.74	Ser	6.50		Lys	5.73	Ser	6.14	Gln	6.62	Thr	7.12
7	Thr	5.59	Lys	5.07	Lys	5.05	Lys	5.24		Gln	5.47	Gln	5.91	Arg	6.62	Gln	6.10
8	Lys	4.74	Tyr	4.44	Thr	4.42	Thr	4.40		Thr	5.03	Thr	5.91	Thr	5.96	Arg	6.10
9	Tyr	4.18	Thr	4.31	Tyr	4.42	Tyr	4.19		Ile	5.03	Gly	4.77	Ile	5.30	Ile	5.08
10	Ile	3.75	Ile	4.06	Gly	3.79	Pro	3.77		Gly	4.41	Ile	4.55	Gly	4.64	Gly	4.75
11	Sup	3.28	Asn	3.55	Pro	3.79	Asn	3.56		Tyr	3.70	Asn	3.18	Tyr	3.97	Trp	4.07
12	Cys	3.07	Gly	3.30	Asn	3.37	Gly	3.56		Glu	3.53	Trp	3.18	Glu	3.31	Tyr	3.73
13	Trp	2.99	Glu	3.04	Glu	3.37	Glu	3.35		Sup	3.35	Tyr	2.95	Trp	3.31	Glu	3.39
14	Gly	2.77	Pro	2.66	Ile	3.37	Ile	3.35		Trp	3.17	Glu	2.95	Pro	2.98	Pro	3.05
15	Asn	2.73	Trp	2.66	Cys	2.74	Phe	2.94		Cys	3.09	Cys	2.95	Cys	2.98	Cys	3.05
16	Glu	2.60	Cys	2.41	Phe	2.74	Cys	2.73		Asn	2.56	Sup	2.95	Phe	2.65	Asn	2.37
17	Pro	2.09	Phe	2.28	Ala	2.11	Ala	2.10		Phe	2.56	Pro	2.50	Asn	2.32	Phe	2.37
18	Phe	1.83	Sup	2.15	Asp	2.11	Asp	2.10		His	2.29	Phe	2.27	Sup	2.32	Ala	2.03
19	His	1.71	Val	1.90	Trp	1.89	Trp	1.89		Ala	2.29	Ala	2.27	Ala	1.99	Asp	1.69
20	Ala	1.45	Ala	1.65	Val	1.68	Sup	1.47		Pro	2.03	Asp	1.82	Asp	1.66	His	1.69
21	Asp	1.24	Asp	1.65	Sup	1.47	Val	1.47		Asp	1.76	His	1.82	His	1.66	Sup	1.36
22	Val	0.94	His	1.27	His	1.26	His	1.26		Val	0.79	Val	1.36	Val	0.66	Val	0.68
		100.00		100.00		100.00		100.00			100.00		100.00		100.00		100.00

## Data Availability

Interactive 3D protein models of the *Enterococcus faecium* structural orthologs can be viewed at https://alphafold.com (accessed on 11 August 2023) under model IDs A0A132P7M2, A0A132Z4D1, A0A132Z369, and A0A133CLV7. Circular and linear virus genomes are included in Supplementary File S1 through File S4 for the strain, species, genus, and family sets. Circular and linear constructs in the preceding files are discernible by their FASTA header names. Scripting in this project was carried out using the R programming language. Code snippets are available at https://github.com/Werner0/tailed_phages (accessed on 11 August 2023). Long reads and short reads from the study by Chen et al. [15] are hosted by the EMBL-EBI (see Section 2).

## Supplementary Materials

The following supporting information can be downloaded at: https://www.mdpi.com/article/10.3390/v16010134/s1, Table S1: Effect of polishing and the meta flag; Table S2: Pervasive structural variants; Table S3: Cross-reference functional annotations per topology per dereplication level; File S1: Strain set circular and linear viral genomes; File S2: Species set circular and linear viral genomes; File S3: Genus set circular and linear viral genomes; File S4: Family set circular and linear viral genomes; File S5: Long-read summary statistics; File S6: Short-read summary statistics.
